# Diagnostic value of a second-generation super-resolution deep learning–based reconstruction combined with a metal artifact reduction algorithm for pelvic CT

**DOI:** 10.1007/s00256-025-05080-4

**Published:** 2025-11-15

**Authors:** Taku Takaishi, Koichiro Yasaka, Kazuyoshi Miyamoto, Kohei Gotoda, Chiaki Sato, Osamu Abe

**Affiliations:** Department of Radiology, The University of Tokyo Hospital, 7-3-1 Hongo, Bunkyo-Ku, Tokyo, 113-8655 Japan

**Keywords:** CT, Hip, Metal artifact reduction, Reconstruction

## Abstract

**Objective:**

To evaluate the impact of combining a second-generation super-resolution deep learning–based reconstruction (DLR2) with a metal artifact reduction (MAR) algorithm in CT images of patients with metal hip implants by assessing both quantitative metrics and qualitative reader ratings.

**Materials and methods:**

This retrospective study included 40 patients (30 females; age range, 54–93 years) with metal hip implants. Images were reconstructed using DLR2, a first-generation DLR (DLR1), and a conventional hybrid iterative reconstruction (HIR), each combined with MAR. Images without MAR were also reconstructed using DLR2 (DLR2-only). Standard deviations (SDs) of attenuation in the regions of interest (ROI) over the bladder, gluteus maximus muscle, and gluteal fat were recorded. Artifact indices for muscle (AI_muscle_) and fat (AI_fat_) were also calculated. Three radiologists independently assessed the depiction of pelvic structures (femoral artery, bladder, rectum, uterus/prostate), artifact reduction, and diagnostic usability with 5-point scores. For statistical analysis, the Wilcoxon signed-rank test was used with the Holm correction for multiple comparisons.

**Results:**

DLR2 + MAR showed significantly lower SDs than DLR1 + MAR, HIR + MAR, and DLR2-only across all three ROIs (*p* < 0.01–0.02). For both AI_muscle_ and AI_fat_, DLR2 + MAR had the lowest values, though differences with DLR1 + MAR were not significant (*p* = 0.80, 0.11). DLR2 + MAR showed significantly higher scores for the depiction of the femoral artery and rectum compared with other + MAR reconstruction methods (*p* < 0.01–0.04), with no significant differences in the remaining categories (*p* = 0.08–0.91). Inter-rater agreement ranged from 0.67 to 0.77, indicating substantial agreement.

**Conclusion:**

Combining DLR2 and MAR improves image quality and visualization of pelvic structures.

**Supplementary Information:**

The online version contains supplementary material available at 10.1007/s00256-025-05080-4.

## Introduction

Metal artifacts caused by orthopedic implants often pose challenges in routine diagnostic workflows. With an aging population, the number of patients undergoing hip replacement surgery is steadily increasing [1]. However, these metallic implants frequently cause streak artifacts on CT images, particularly pelvic CT, which is the focus of this study. These artifacts arise from a phenomenon known as photon starvation, where the metal significantly attenuates the X-ray beam, resulting in insufficient photon counts reaching the detector and ultimately causing reconstruction errors [2].

To address this issue, metal artifact reduction (MAR) algorithms have been developed by multiple CT vendors and are already in clinical use [3]. These MAR algorithms generally follow a similar underlying principle. First, metal components are segmented from the original image. Then, the metal-only image is forward-projected to generate a metal-only sinogram. The metal-affected portions of the sinogram are subsequently replaced with interpolated values derived from the surrounding non-metal data. A final image is reconstructed from the corrected sinogram. MAR has shown promising results in image quality improvement in CT images with metal hip implants [4–7].

Around the 2020 s, deep learning–based reconstruction (DLR) algorithms began to be implemented in commercial CT scanners. DLR is particularly noteworthy for its ability to reduce image noise [8], contributing to improved lesion depiction and detection performance without extending reconstruction time [9–11], unlike iterative reconstruction methods [12]. Recently, a second-generation super-resolution DLR (DLR2) has emerged. This software, cleared by the U.S. Food and Drug Administration in 2024 [13], was trained using high–spatial resolution CT images [14], enabling both noise reduction and enhanced spatial resolution. The application of this super-resolution DLR has been investigated in various fields [15–17]. When combined with MAR’s sinogram-based artifact correction, DLR2 is expected to further reduce residual noise and improve visualization of fine structures. However, the combined effect of DLR2 and MAR has not yet been systematically evaluated.

This study aimed to evaluate the impact of combining DLR2 with MAR in CT images of patients with metal hip implants by assessing both quantitative metrics, including standard deviation (SD) and artifact index (AI), and qualitative reader ratings.

## Materials and methods

### Patient selection

This study was approved by the institutional review board, and the requirement for informed consent was waived due to its retrospective design. The inclusion criteria were consecutive patients with metallic implants in the hip joints who underwent CT between February 2025 and July 2025 using a dedicated scanner. The exclusion criteria were contrast delay > 150 s (as excretion of contrast media may compromise bladder depiction) and raw data missing.

### CT protocols

All the patients underwent CT examination using Aquilion ONE INSIGHT Edition (Canon Medical Systems, Otawara, Japan). The CT scanning parameters were the following: tube voltage, 120 kVp; tube current, automatic tube current modulation was used with the standard deviation (SD) set at 13.0; beam pitch, 0.813; and gantry rotation time, 0.5 s. When contrast media were used, the concentration and volume were adjusted based on body weight (600 mgI/kg), with a scan delay of 90–120 s after injection.

### Metal artifact reduction

**Fig. 1:**
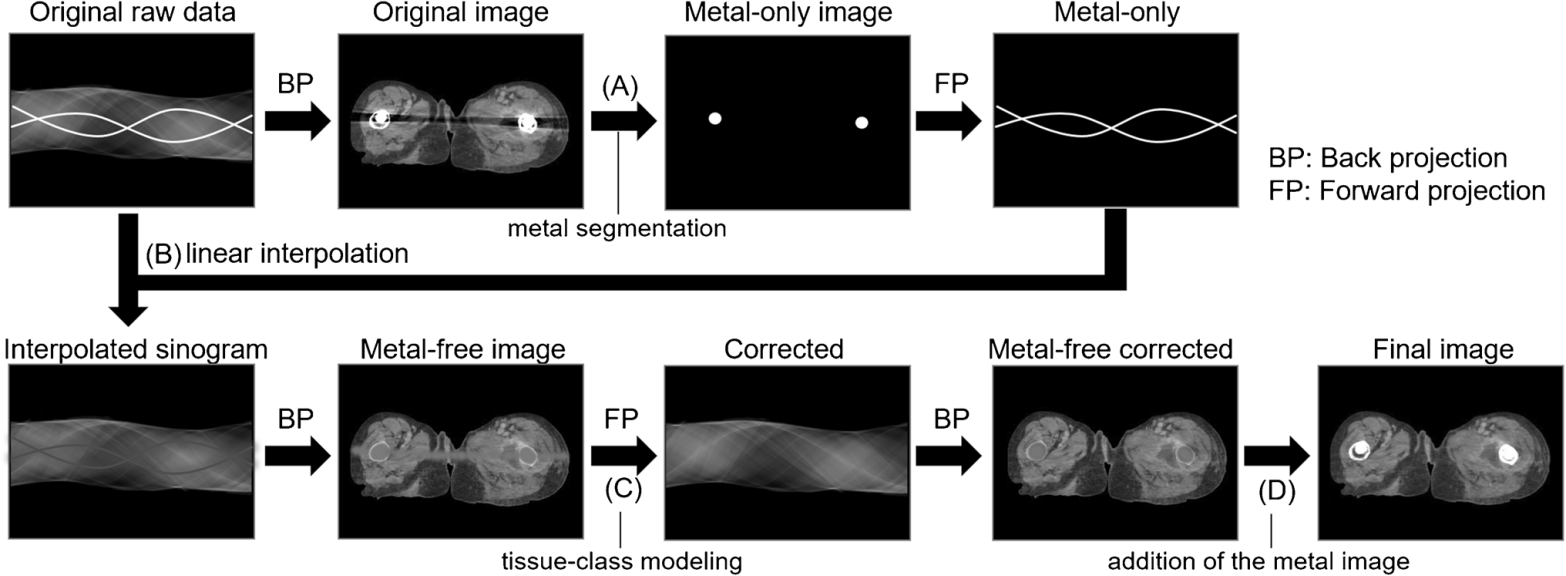
Flow diagram of the single-energy metal artifact reduction (SEMAR) algorithm. First, the metal is segmented on the original image using a threshold approach (**A**), and the metal-only image is forward projected to obtain a metal-only sinogram. Next, the metal-blocked part in the sinogram is linearly interpolated using the neighboring non-metal-blocked measurements (**B**). Then, the interpolated sinogram is back projected, and the resulting metal-free image is classified into “air,” “water,” and “bone” by threshold differentiation to obtain a prior image. This prior image is forward projected onto the metal trace to provide a corrected sinogram (**C**). Finally, the corrected sinogram is back projected and the metal-only image is added (**D**)

As an MAR algorithm, we used single-energy metal artifact reduction (SEMAR) (Canon Medical Systems). It identifies and segments the distorted data projection resulting from metallic implants and then modifies these data by replacing them with approximations of the corrected values using repetitive forward and backward projections and linear interpolation [6, 18]. The detail of the SEMAR algorithm is shown in Fig. 1.

### Image reconstruction

We used two DLR algorithms developed by Canon Medical Systems as follows: DLR1 (AiCE, Advanced Intelligent Clear-IQ Engine), DLR2 (PIQE, Precise IQ Engine). DLR1 is a first-generation DLR algorithm, which was designed to distinguish signal features from noise. It was trained on clinical reference images acquired using a high tube current and reconstructed using a model-based iterative reconstruction (accounting for modelling of system optics, system physics, scanner statistical properties, and human anatomy) [19]. DLR2 is a second-generation super-resolution DLR algorithm which was built upon the foundation of DLR1 to enhance spatial resolution and preserve signal features under standard clinical scanning conditions. It was trained with reference images from the ultra-high-resolution CT scanner (0.25-mm slice thickness) [20]. In addition, we used a conventional hybrid iterative reconstruction (HIR) algorithm (Adaptive Iterative Dose Reduction 3D, Canon Medical Systems).

For image analyses, we compared four reconstruction methods as follows: DLR2 with MAR (DLR2 + MAR), DLR1 with MAR (DLR1 + MAR), HIR with MAR (HIR + MAR), and DLR2 without MAR (DLR2-only). The following parameters remained consistent across the four methods: slice thickness, 3.0 mm; slice interval, 3.0 mm; field of view, 35–40 cm (adjusted to body size); and *Z*-axis range, from the iliac crest to the ischial tuberosity. The pixel resolution was 1024 × 1024 for DLR2, whereas it was 512 × 512 for DLR1 and HIR, using a vendor-specific body kernel optimized for soft-tissue contrast.

### Quantitative image analysis

A radiologist (T.T. with 9 years of experience) performed the quantitative image analysis using ImageJ (http://imagej.nih.gov/ij), following a previously described method [6, 7]. At the slice where metal artifacts were most prominent, ovoid regions of interest (ROIs) were placed in the bladder, the gluteus maximus muscle, and gluteal fat. ROIs were also placed in the iliopsoas muscle and dorsal subcutaneous fat at the level where metal artifacts were absent. The bladder ROI was drawn as large as possible, avoiding contrast media excretion if present. The remaining four ROIs were each drawn with a size of at least 100 mm^2^ and placed on the ipsilateral side of the implants (the right side was chosen for bilateral implants). The ROI copy-and-paste function was used to ensure that the location and size of each ROI were identical across different algorithms. Standard deviations (SDs) of attenuations in these ROIs were recorded to evaluate overall image noise and artifacts.

In addition, an artifact index (AI) was calculated to quantify the artifact component because SD inherently reflects both image noise and metal-induced artifacts. As previously described [21], the AI isolates the artifact component by subtracting the background noise from artifact-free regions. Specifically, AI_muscle_ and AI_fat_ were calculated as follows:

$${AI}_{\mathrm{muscle}}=\sqrt{{{SD}_{\mathrm{gluteus}}}^{2}-{{SD}_{\mathrm{iliopsoas}}}^{2}}$$

$${AI}_{\mathrm{fat}}=\sqrt{{{SD}_{\mathrm{gluteal}}}^{2}-{{SD}_{\mathrm{dorsal}}}^{2}}$$

Here, SD_gluteus_, SD_iliopsoas_, SD_gluteal,_ and SD_dorsal_ refer to SDs of ROIs in the gluteus maximus muscle, iliopsoas muscle, gluteal fat, and dorsal subcutaneous fat, respectively. Lower AI corresponds to better artifact reduction.

### Qualitative image analysis

Three radiologists (C.S, K.G, and K.M. with 6, 4, and 2 years of experience, respectively) independently assessed the depiction of 4 pelvic structures (femoral artery, bladder, rectum, uterus/prostate) with 5-point metrics (5, fully visualized; 4, mostly clear; 3, moderately clear; 2, blurred; 1, unrecognizable). They also evaluated artifact reduction (5, no artifact; 4, slight; 3, moderate; 2, severe in small area; 1, severe in large area) and diagnostic usability (5, excellent; 4, good; 3, fair; 2, suboptimal; 1, nondiagnostic). All images were reviewed using the ImageJ software. The reconstruction method for each image was blinded to the readers, and the display order was randomized.

### Statistical analysis

Statistical analyses were performed using R software (version 4.5.1) [22]. For the quantitative analysis, SDs of the ROIs and AIs were compared using the Wilcoxon signed-rank test, as normality was not satisfied on the Shapiro–Wilk test. For the qualitative analysis, readers’ ratings for each reconstruction method were compared using the Wilcoxon signed-rank test, and the inter-rater agreement among the three radiologists was assessed using Kendall’s coefficient of concordance (Kendall’s *W)*. When performing multiple comparisons, the Holm correction was applied. A two-sided *p*-value of < 0.05 was considered statistically significant.

## Results

### Patient selection

After excluding cases with contrast delay > 150 s (*n* = 2) and raw data missing (*n* = 1), 40 patients were included in the final analysis (Table 1). The implant material was determined from medical records; among the cases with available information, all identified implants (21/21) were primarily composed of titanium (see Supplemental Material S1). Regarding the radiation exposure, the median dose-length product was 8.0 mGy·cm (interquartile range 6.2–10.9 mGy·cm), and the median CT dose index volume was 573 mGy (interquartile range 411–770 mGy).

**Table 1:** Patient demographics

Patient	*n* = 40
Age, mean (range)	73.0 (54–93)
Sex (male to female)	10:30
Implant type	IF 8, BHA 11, THA 21
Laterality	Right 18, left 16, bilateral 6*
Contrast media	With 33, without 7

*IF* internal fixation, *BHA *bipolar hip arthroplasty, *THA* total hip arthroplasty. *All patients with bilateral implants received THA

### Quantitative image analysis

**Fig. 2:**
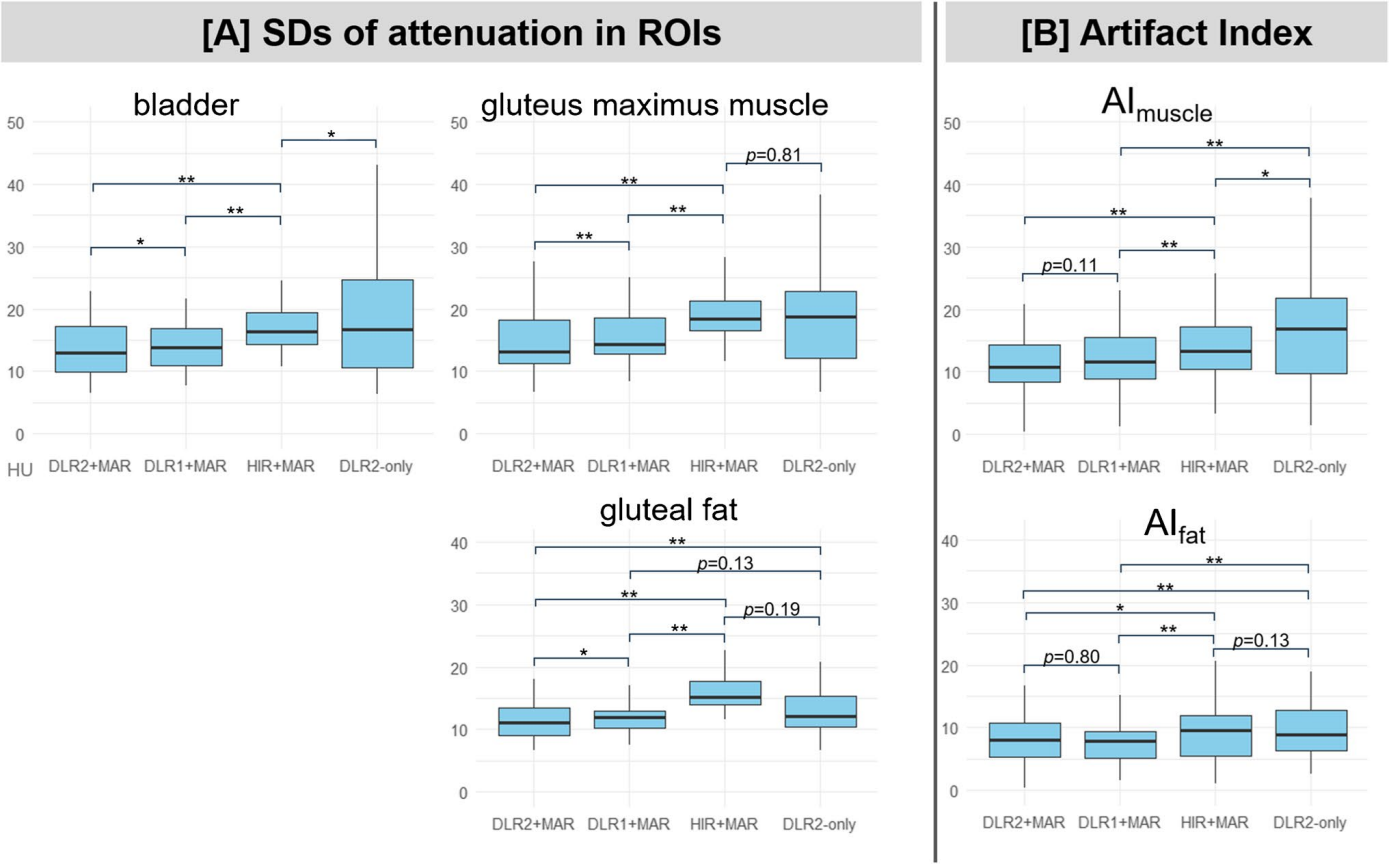
Result of the quantitative analysis. The boxes indicate the interquartile range (IQR) with the median represented by the central line; whiskers extend to the most extreme data points within 1.5 × IQR. *p = 0.01–0.05; **p < 0.01. **A** Standard deviation (SD) of the attenuation value (Hounsfield unit, HU) in the ovoid region of interest (ROI) over the bladder, gluteus maximus muscle, and gluteal fat. **B** Artifact indices (AIs) reflect the differences in SDs between metal-artifact-affected and metal-artifact-free regions, with lower AIs indicating better artifact reduction. AI_muscle_ represents the SD difference between the metal-affected muscle (gluteus maximus muscle) and metal-artifact-free muscle (iliopsoas muscle). AI_fat_ represents the SD difference between the metal-affected fat (gluteal fat) and metal-artifact-free fat (dorsal fat)

The SDs of attenuation in ROIs are shown in Fig. 2A. In the metal-artifact-affected regions (bladder, gluteus maximus muscle, and gluteal fat), DLR2 + MAR showed significantly lower SDs compared with other methods. In the metal-free regions (iliopsoas muscle and dorsal fat), DLR2 + MAR showed significantly lower SDs compared with DLR1 + MAR and HIR + MAR. However, the difference in SD between DLR2 + MAR and DLR2-only was marginal, with a median SD of 6.61 and 6.64 (*p* = 0.049) in the iliopsoas muscle, and 6.57 and 6.58 (*p* = 0.040) in the dorsal fat. In both AI_muscle_ and AI_fat_ (Fig. 2B), DLR2 + MAR demonstrated the lowest values across all reconstruction methods, with statistically significant reductions observed compared with HIR + MAR and DLR2-only. A consistent trend was observed when comparing DLR2 + MAR and DLR1 + MAR, with a median AI_muscle_ of 10.6 and 11.6 (*p* = 0.11) and a median AI_fat_ of 7.94 and 7.95 (*p* = 0.80).

### Qualitative image analysis

**Fig. 3:**
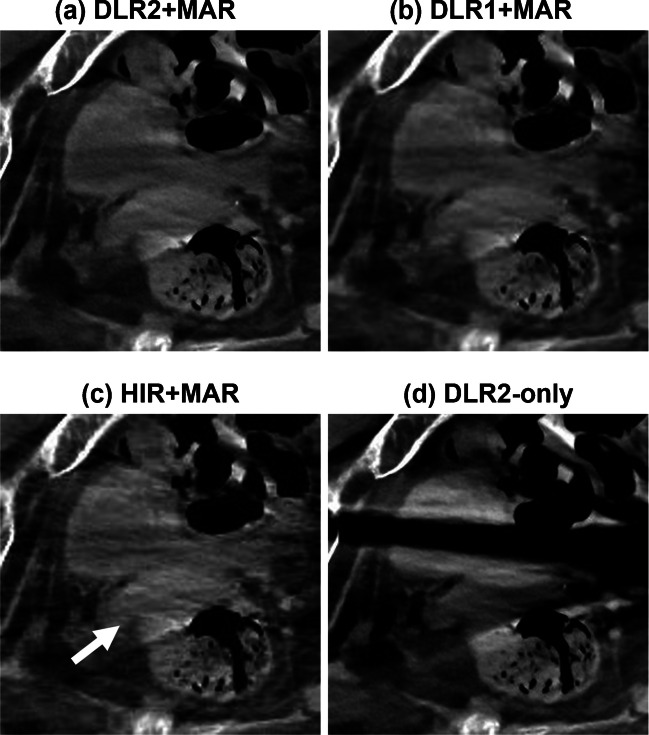
Visualization of the bladder, uterus, and rectum (93-year-old female with bilateral hip prostheses). The *white arrow* indicates the uterus, which is located between the bladder and rectum. **a** to **c** succeed in suppressing the metal artifact, while **d** shows a pronounced dark band artifact over the bladder. **a** shows reduced intravesical noise and clearer delineation of the uterus and rectum compared with **b** and **c**. The average scores given by three raters for each organ depiction in images (**a**, **b**, **c**, **d)** were as follows: bladder (3.0, 2.3, 2.3, 1.0), uterus (3.3, 2.7, 3.0, 1.3), and rectum (3.0, 3.0, 3.0, 2.7)

**Fig. 4:**
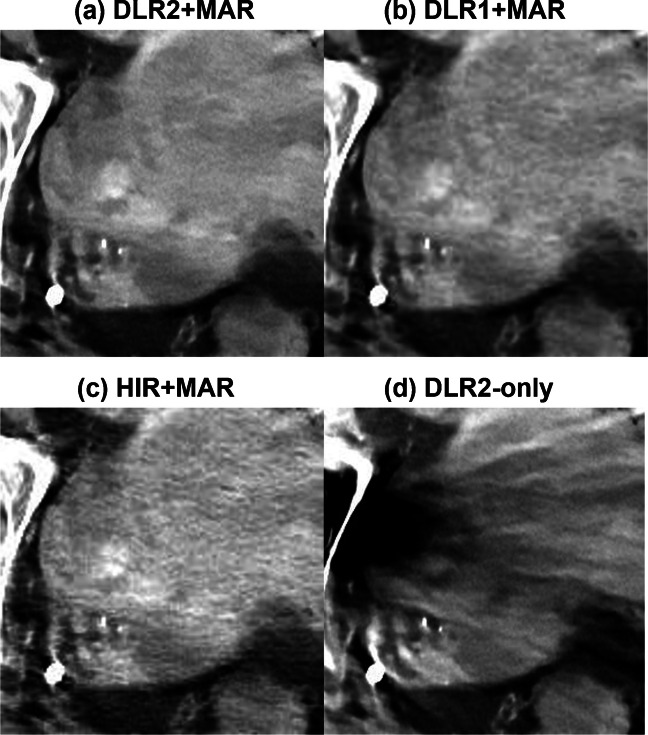
Depiction of a uterine leiomyoma (68-year-old female with right hip prosthesis). Compared with **c**, **b** shows reduced noise. **a** shows increased sharpness and provides better resolution of the degenerative changes within the leiomyoma. Severe metal artifact degrades lesion depiction in **d**

**Fig. 5:**
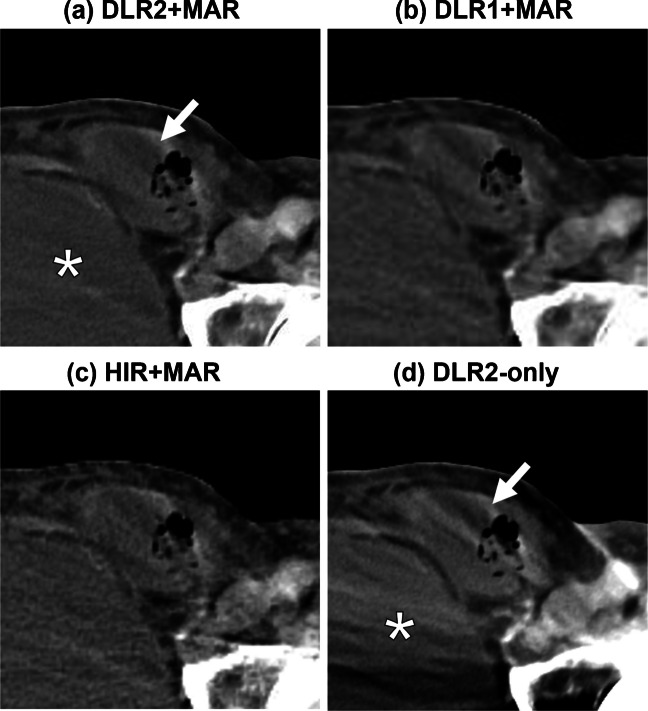
Incarcerated small bowel in a left inguinal hernia (77-year-old male with left hip prosthesis). Both **a** and **d** provide a clearer delineation of the bladder (*asterisk*) and the herniated small bowel (*white arrow*) compared with **b** and **c**. This likely reflects the fact that DLR2 was trained to preserve higher spatial resolution. Streak artifacts from the metal implant remain in **d**

Results of the qualitative analysis are summarized in Table 2. DLR2 + MAR showed the highest scores in all categories, while statistically significant differences against DLR1 + MAR and HIR + MAR were observed in two categories (femoral artery and rectum). Inter-rater agreement was substantial (0.61–0.80) in all the categories, according to the Landis and Koch criteria [23]. Examples of reconstructed CT images are shown in Figs. 3, 4, and 5.

**Table 2:** Results of the qualitative image analysis

	Average Rating (mean of three raters)				Comparison between methods				Inter-rater
	(a)	(b)	(c)	(d)	(a) vs. (b)	(a) vs. (c)	(b) vs. (c)	(a) vs. (d)	agreement
	DLR2+MAR	DLR1+MAR	HIR+MAR	DLR2 only	*p* value				Kendall's *W*
femoral artery	3.13	2.98	2.92	2.43	**< 0.01**	**< 0.01**	0.20	**< 0.01**	0.73
bladder	3.32	3.24	3.25	2.42	0.31	0.34	0.87	**< 0.01**	0.70
rectum	3.62	3.52	3.52	2.99	**0.01**	**0.04**	0.84	**< 0.01**	0.67
uterus/prostate	3.43	3.43	3.39	2.61	0.91	0.87	0.87	**< 0.01**	0.71
artifact reduction	3.12	3.09	3.12	1.91	0.69	0.78	0.71	**< 0.01**	0.68
diagnostic usability	3.21	3.15	3.13	2.30	0.15	0.08	0.63	**< 0.01**	0.77

A fully detailed description of each reader’s scores is provided in Supplemental Table S2. DLR, deep learning-based reconstruction; HIR, hybrid iterative reconstruction; MAR, metal artifact reduction. The bold text in the table indicates statistically significant differences

## Discussion

In this study, we investigated whether a second-generation super-resolution deep learning–based reconstruction (DLR2) with a metal artifact reduction (MAR) algorithm can improve image quality and the visualization of pelvic structures in CT images of patients with metal hip implants. We compared DLR2 + MAR with other reconstruction methods including a first-generation DLR (DLR1) + MAR, a conventional hybrid iterative reconstruction (HIR) + MAR, and DLR2 without MAR (DLR2-only), using both quantitative and qualitative assessments.

Various studies have investigated metal artifact reduction methods, including acquisition parameter optimization, dual-energy CT, and end-to-end deep learning–based MAR techniques [3]. While basic acquisition optimization (high tube current, high tube voltage, and lower pitch) can suppress artifacts, it involves a higher risk of ionizing radiation. Dual-energy CT is highly effective but operationally complex, requiring pre-scan settings for two energy levels. In contrast, MAR used in this study offers practical advantages as it can be applied retrospectively to images already acquired. Although end-to-end deep learning–based MAR is an active area of research [24–26], it has not yet been implemented in clinical CT systems. So far, few studies have examined the integration of commercially available DLR with MAR. One previous study investigated a first-generation DLR (DLR1 = AiCE) with MAR for hip implants [27]. Our study is unique in investigating the clinical potential of a second-generation super-resolution DLR (DLR2 = PIQE) combined with MAR.

In the quantitative analysis, DLR2 + MAR yielded the lowest SDs in all artifact-affected ROIs (bladder, gluteus maximus muscle, and gluteal fat) (Fig. 2A). However, as DLR algorithms are inherently effective at reducing stochastic image noise [28], lower SDs do not necessarily indicate true artifact suppression. Therefore, we employed artifact indices (AI_muscle_ and AI_fat_), previously used in metal artifact studies [29, 30], which measure the balance of SD between an artifact-affected region and an artifact-free counterpart. This approach showed that both DLR2 + MAR and DLR1 + MAR outperformed HIR + MAR (Fig. 2B). This result demonstrates DLR’s superiority over conventional HIR in mitigating both stochastic noise and residual metal artifacts. Residual metal artifacts are attenuation fluctuations that remain after MAR processing and are occasionally caused by scatter and edge effects [31].

Although AI_muscle_ and AI_fat_ did not differ significantly between DLR2 + MAR and DLR1 + MAR, the design of these two DLR algorithms differs. DLR1 (AiCE) was primarily optimized for noise suppression, whereas DLR2 (PIQE) was trained using ultra-high-resolution CT data (0.25-mm slice thickness) to enhance spatial resolution while maintaining denoising performance [20]. Previous studies have shown the superiority of DLR2 in visualizing fine anatomical structures, such as coronary in-stent restenosis [32] and adrenal glands [33]. Consistent with these reports, our qualitative analysis demonstrated that DLR2 + MAR outperformed DLR1 + MAR in depicting small-caliber structures (e.g., femoral artery) and regions with irregular margins (e.g., rectum) (Table 2), underscoring its potential clinical advantage in detecting subtle lesions in pelvic CT.

The effects of MAR varied by anatomical regions. In the bladder and gluteus maximus muscle, + MAR reconstructions showed lower SDs than DLR2-only (Fig. 2A). Conversely, in the gluteal fat, SD reduction with DLR2-only surpassed HIR + MAR, although the difference was not statistically significant. This pattern aligns with the findings of Chen et al. [7], who reported that SEMAR reduced bladder SD by 64% (from 39.6 to 14.3 HU) but subcutaneous fat SD by only 38% (from 34.6 to 21.3 HU). A possible explanation is that gluteal fat is relatively distant from hip implants and thus less affected by metal artifacts. Indeed, in Fig. 2A, the whisker (representing the data range) of DLR2-only for gluteal fat is shorter than that for the gluteus maximus muscle (located closer to the hip implant) or bladder (which is prone to strong dark band artifacts caused by bilateral hip implants, as exemplified in Fig. 3). 

Regarding radiation exposure, the CT scans in this study were acquired using standard dose protocols. As indicated in the demographic table (Table 1), the population receiving hip implants tends to be older, rendering the relative impact of radiation exposure less clinically significant. Nevertheless, previous studies have shown that DLR allows for 25% and 43% dose reduction for head CT and coronary CT, respectively, while preserving image quality [15, 34]. Future research should explore the extent to which radiation dose can be lowered when combining DLR and MAR.

This study has several limitations. First, the retrospective design may introduce selection bias. However, the encouraging findings support the need for prospective validation with a larger cohort. Second, the sample size was limited, precluding subgroup analysis by implant type, implant laterality (unilateral vs. bilateral), or use of contrast media (with vs. without). Third, due to the difficulty of identifying patients with specific pathologies who also had metal hip prostheses, lesion detection performance was not assessed. Fourth, because all images were reconstructed using a body kernel, evaluation of the bone–implant interface, such as the femur and acetabulum, was not included. Fifth, while similar advanced DLR algorithms are available from other vendors, their detailed mechanisms differ. Therefore, the results of this study cannot be directly applied to CT data obtained with scanners from other vendors.

In conclusion, DLR2 + MAR showed favorable results in both quantitative and qualitative analyses. This combination enhances the visualization of fine pelvic structures in CT images of patients with metal hip implants, thereby facilitating a more accurate assessment of pelvic anatomy and improving diagnostic confidence.

## Supplementary Information

Below is the link to the electronic supplementary material.

ESM 1 (DOCX 27.8 KB)

## Data Availability

Due to the nature of this research, patients of this study did not agree for their data to be shared publicly, so supporting data is not available.
