# Supplementary material for: Diagnostic value of a second-generation super-resolution deep learning–based reconstruction combined with a metal artifact reduction algorithm for pelvic CT
Source: Skeletal Radiol. 2025 Nov 15;55(4):757–65. doi: 10.1007/s00256-025-05080-4 (PMC12891154; doi:10.1007/s00256-025-05080-4)
Supplement: Supplementary file 1 — (DOCX 27.8 KB) [file 256_2025_5080_MOESM1_ESM.docx]

**S1. Types of Hip Implant**

product name, (company), main metal component, number of implants

>Internal Fixation, n=8

Prima Hip Screw, (Japan MDM), titanium, n=2

Inter TAN, (Smith&Nephew), titanium, n=2

Unknown, n=4

>Bipolar Hip Arthroplasty n=11

BiCONTCT, (Aesculap), titanium, n=2

GMRS, Stryker, titanium, n=1

Unknown, n=8

>Total Hip Arthroplasty, n=27 from 21 patients (including 6 patients with bilateral implants)

SQRUM/INITIA, (Kyocera), titanium, n=8

SQRUM/Jtaper, (Kyocera), titanium, n=3

R3＋SL PLUS OXINIUM, (Smith&Nephew), titanium, n=2

GS Cup + Optimys, (Nakashima), titanium, n=1

Unknown, n=13

**S2. Detailed Distribution of Each Reader’ Rating**

|  | Reader experience | DLR2 +MAR | DLR1 +MAR | HIR +MAR | DLR2  only |  | Inter-rater  agreement |
| --- | --- | --- | --- | --- | --- | --- | --- |
| femoral artery | 6 yrs | 7/4/28/1/0 | 1/10/24/5/0 | 2/7/26/5/0 | 8/0/13/15/4 |  |  |
|  | 4 yrs | 5/5/10/18/2 | 4/4/11/17/4 | 3/6/8/20/3 | 6/1/2/11/20 |  |  |
|  | 2 yrs | 4/6/22/8/0 | 4/3/25/8/0 | 2/6/21/11/0 | 4/3/7/18/8 |  |  |
| mean±SD |  | 3.13±0.97 | 2.98±0.91 | 2.92±0.89 | 2.43±1.33 |  | 0.27 |
| bladder | 6 yrs | 4/3/21/11/0 | 1/6/22/10/0 | 1/5/21/12/0 | 4/3/2/23/7 |  |  |
|  | 4 yrs | 8/19/12/0/0 | 6/21/12/0/0 | 8/19/12/0/0 | 6/2/11/14/6 |  |  |
|  | 2 yrs | 2/3/29/5/0 | 2/2/26/9/0 | 1/4/27/7/0 | 3/4/1/22/9 |  |  |
| mean±SD |  | 3.32±0.86 | 3.24±0.82 | 3.25±0.83 | 2.42±1.21 |  | 0.21 |
| rectum | 6 yrs | 4/9/25/2/0 | 1/8/27/4/0 | 2/7/24/7/0 | 5/3/19/12/1 |  |  |
|  | 4 yrs | 6/17/15/2/0 | 7/19/12/2/0 | 5/26/7/2/0 | 6/3/16/13/2 |  |  |
|  | 2 yrs | 8/17/14/1/0 | 7/12/20/1/0 | 9/9/20/2/0 | 5/3/23/7/2 |  |  |
| mean±SD |  | 3.62±0.79 | 3.52±0.79 | 3.52±0.84 | 2.99±1.02 |  | 0.28 |
| uterus/prostate | 6 yrs | 3/4/18/7/0 | 0/8/16/8/0 | 1/5/17/9/0 | 4/3/7/16/2 |  |  |
|  | 4 yrs | 6/13/11/2/0 | 5/22/3/2/0 | 5/17/9/1/0 | 6/2/7/9/8 |  |  |
|  | 2 yrs | 4/9/19/1/0 | 5/6/18/4/0 | 4/8/20/2/0 | 5/1/11/8/11 |  |  |
| mean±SD |  | 3.43±0.85 | 3.43±0.87 | 3.39±0.83 | 2.61±1.30 |  | 0.23 |
| artifact reduction | 6 yrs | 0/7/27/6/0 | 0/7/27/6/0 | 0/7/28/5/0 | 0/5/1/27/7 |  |  |
|  | 4 yrs | 0/10/24/6/0 | 0/8/26/6/0 | 0/10/25/5/0 | 0/1/4/12/23 |  |  |
|  | 2 yrs | 0/12/26/2/0 | 0/8/32/0/0 | 0/7/33/0/0 | 0/5/1/25/9 |  |  |
| mean±SD |  | 3.12±0.59 | 3.09±0.53 | 3.12±0.52 | 1.91±0.86 |  | 0.51 |
| diagnostic usability | 6 yrs | 5/9/15/11/0 | 1/10/17/12/0 | 2/8/14/16/0 | 6/2/0/27/5 |  |  |
|  | 4 yrs | 3/7/23/7/0 | 1/14/21/4/0 | 3/13/20/4/0 | 5/2/2/22/9 |  |  |
|  | 2 yrs | 3/9/25/2/1 | 3/7/23/7/0 | 1/10/22/7/0 | 4/3/1/20/12 |  |  |
| mean±SD |  | 3.21±0.86 | 3.15±0.77 | 3.13±0.82 | 2.30±1.23 |  | 0.50 |

Counts for each score (5/4/3/2/1) are presented. Inter-rater agreement among the three readers was assessed using Kendall’s coefficient of concordance (Kendall’s W). Note that the sum of each score does not always equal the total number of images (n = 40) because the target organ was outside the scan range or resected in some patients.
